# Lemon myrtle extract enhances muscle hypertrophy induced by low-load bodyweight resistance training in older adults: Findings from two independent randomized controlled trials

**DOI:** 10.1016/j.jnha.2025.100706

**Published:** 2025-10-11

**Authors:** Shuji Sawada, Azusa Nishino, Shinichi Honda, Yuji Tominaga, Shiori Makio, Hayao Ozaki, Shuichi Machida

**Affiliations:** a Faculty of Health and Sports Science, Juntendo University, Chiba, Japan; b Kaneka Corporation, Osaka, Japan; c Graduate School of Medicine, Juntendo University, Tokyo, Japan; d Department of Sport and Health Science, Tokai Gakuen University, Aichi, Japan; e Graduate School of Health and Sports Science, Juntendo University, Chiba, Japan

**Keywords:** Sarcopenia, Resistance training, Muscle hypertrophy, Plant extract, Lemon myrtle

## Abstract

**Objectives:**

Previous literature has shown that combining lemon myrtle (LM) leaf extract with low-load resistance training may promote muscle hypertrophy. The current our studies aimed to verify the effects of LM intake combined with different training volumes on anterior thigh (AT) muscle thickness in older adults.

**Design:**

Two independent randomized, double-blind, placebo-controlled trials.

**Setting:**

Community-based training intervention program.

**Participants:**

A total of 125 Japanese adults aged ≥65 years, with self-reported declines in muscle strength or walking ability. Study 1 (n = 47; LM group: n = 25, placebo group: n = 22) and Study 2 (n = 41; LM group: n = 22, placebo group: n = 19) were conducted independently, each with separate randomization into LM and placebo groups.

**Intervention:**

Both studies involved low-load bodyweight resistance training twice weekly for 12 weeks. Study 1 compared LM + three sets of training with placebo + three sets; whereas Study 2 compared LM + one set with placebo + one set.

**Measurements:**

AT muscle thickness (primary outcome) was assessed using B-mode ultrasound at baseline, 6, and 12 weeks. Secondary outcomes were normal walking speed (10-m walk test, m/s), maximum walking speed (10-m walk test, m/s), and 30-second chair stand (CS-30, repetitions).

**Results:**

In Study 1, the LM + exercise group showed a greater increase in AT muscle thickness than the placebo + exercise group (12-week difference: 1.29 mm; 95% CI: −0.17 to 2.75 mm), although the difference was not statistically significant. In Study 2, the LM + exercise group showed a significant increase in AT muscle thickness compared with the placebo + exercise group (1.59 mm; 95% CI: 0.19–2.98 mm). No significant group-by-time interaction was found for secondary outcomes in either study.

**Conclusions:**

LM intake may enhance muscle hypertrophy when combined with low-load resistance training in older adults with self-reported declines in muscle strength decline, particularly under low-volume training conditions. Further studies are needed to establish its clinical relevance.

## 1 Introduction

Population aging is a major social challenge worldwide. Maintaining skeletal muscle mass and function is essential for healthy aging, as skeletal muscles play important roles in physical functions related to activities of daily living [1] and whole-body energy regulation, mainly through glucose and lipid metabolism [2], thereby contributing to homeostasis. However, muscle mass and strength decline with age [3,4], a condition known as sarcopenia, which is associated with various chronic diseases [[5], [6], [7], [8]], movement disorders [9,10], and mortality [11]. Recognized in the International Classification of Diseases, 10th Revision (ICD-10, code M62.84) [12], sarcopenia has become an important target for prevention and treatment.

Updated guidelines from the European and Asian working groups [13,14] recommend exercise combined with adequate nutrition, particularly sufficient protein intake, to maintain muscle mass and function [15]. Previous studies have shown that such combined interventions, whether in healthy older adults [16] or in those meeting the Asian Working Group for Sarcopenia (AWGS 2014) criteria [[17], [18], [19]], can improve muscle strength and function, although the effects on muscle mass vary.

Lemon myrtle (LM; *Backhousia citriodora*) leaf extract, which possesses antimicrobial [20,21], anti-inflammatory [[22], [23], [24]], and antioxidant activities [[23], [24], [25], [26]], has been investigated as a potential agent for muscle hypertrophy. Our previous study demonstrated that LM extract and its active compound, casuarinin, activated skeletal muscle satellite cells (SCs) both in vitro and in vivo [27]. SCs are essential for muscle regeneration after injury or exercise, and their functional decline contributes to sarcopenia [28]. In a pilot study in older adults, LM leaf extract combined with typical-volume (three-set) low-load resistance training using body weight produced a greater increase in muscle size than resistance training alone [29], suggesting that LM ingestion may enhance hypertrophic adaptation to resistance training.

Resistance training volume is generally associated with hypertrophy in a dose–response manner [30], but the optimal volume for older adults, particularly those with sarcopenia or frailty, is unclear. Low-volume programs using only body weight could improve accessibility and adherence if effective.

The current our studies aimed to: (1) confirm whether the effects of LM, observed in our pilot study with typical-volume training, could be replicated in a larger sample of older adults with self-reported muscle strength decline (Study 1); and (2) determine whether LM enhances the effects of low-volume (one-set) training (Study 2). We report the results of two independent randomized controlled trials conducted separately, each comparing LM with placebo.

## 2 Materials and methods

### 2.1 Participants

Older Japanese men and women were recruited through a Contract Research Organization. When recruiting participants, the inclusion criteria were: "Age 65 or older, and awareness of reduced muscle mass or strength and/or reduced walking speed." Accordingly, the present assessment included individuals who subjectively perceived declines in their muscle mass, strength, and/or walking speed, which was determined through self-assessment. Participants were excluded from the study if they met the following criteria: (1) taking antidiabetic medications; (2) medically prohibited from exercising; (3) experiencing symptoms that interfered with training, such as lower back pain or arthralgia; (4) regularly consuming supplements, functional foods, or medications intended to improve muscle or arthrosis symptoms; (5) daily attendance at a health club; (6) presenting with considerable undernutrition; and (7) judged ineligible by a doctor. Appropriate explanations were provided to participants regarding important factors, including the study purpose, content, methods, and predicted adverse reactions. Informed consent was obtained from all participants in writing.

We conducted the first screening (medical examination, blood test, and questionnaire survey) on 327 participants who provided informed consent. We excluded 108 participants, mainly for the following reasons: arrhythmia, shortness of breath, a medical history of heart disease, high blood pressure, obesity (body mass index [BMI] > 30 kg/m^2^), possible diabetes (high blood sugar and hemoglobin A1c levels), underweight (BMI < 18.5 kg/m^2^), and regular exercise habits. We selected 219 participants for the second screening. The participants underwent a second screening involving body composition analysis, normal and maximum walking speed measurements, and the 30-s chair stand (CS-30) test. Participants with a maximum walking speed of ≥2.2 m/s or a CS-30 score of ≥25 were excluded because they did not exhibit low values in physical function or muscle strength.

In total, 125 Japanese men and women (70.6 ± 4.5 years) were selected for participation based on the aforementioned criteria. Participants in Study 1 were randomized into two groups (LM + exercise and placebo + exercise) separately from participants in Study 2, who were also randomized into two groups (LM + exercise and placebo + exercise). There was no overlap in participants across the studies, and each study had independent randomization and intervention periods. Both studies were conducted from July 3, 2023, to December 31, 2023. Randomization in each study was stratified by allocation factors: age, sex, maximum walking speed, and CS-30 score.

### 2.2 Study design

To evaluate the efficacy of LM extract, two randomized double-blind placebo-controlled intervention studies (Studies 1 and 2) were designed. All the experiments conformed to the Declaration of Helsinki and were conducted under the supervision of the principal investigator. The study protocol and related documents were approved by the Ethics Committee of the Juntendo University Graduate School of Health and Sports Science Institutional Review Board (2022−131). These studies were registered in the UMIN Clinical Trials Registry (UMIN 000050432). The studies were conducted between May 2023 and December 2023 in Tokyo, Japan.

In the 12-week randomized controlled intervention trial, participants were randomly categorized into two studies, each with two groups: typical-volume (three-set) low-load resistance training twice per week with placebo intake (placebo + exercise group) or LM intake (LM + exercise group) for Study 1 and low-volume (one set) low-load resistance training twice per week with placebo + exercise group or LM + exercise group for Study 2. The researchers who assessed each parameter were blinded to group allocation. Body composition, anterior thigh (AT) muscle thickness, normal and maximum walking speeds, and CS-30 scores were assessed at baseline (0 weeks), mid-intervention (6 weeks), and post-intervention (12 weeks). The primary endpoint in both Studies 1 and 2 was muscle thickness. The resistance training programs for Studies 1 and 2 were conducted by experienced instructors at the training gymnasiums of Sports Oasis Inc. The experienced instructors were trained in instructional methods under their supervisor’s guidance. Additionally, the Brief-type Self-administered Diet History Questionnaire (BDHQ) was administered at 0, 6, and 12 weeks to assess changes in diet quality and quantity.

All participants were instructed to engage in a low-load resistance training program using body weight and to avoid changing their dietary patterns and lifestyle habits throughout the intervention period.

The participants began receiving tablets on the measurement day during week 0 and received the resistance training program according to a specified schedule.

### 2.3 Resistance training

In each experiment (Studies 1 and 2), the resistance training program was conducted twice a week for 12 weeks by proficient sports instructors from the training gymnasiums of Sports Oasis Inc. at three sites: the Yukigaya branch (Mondays and Thursdays), the Musashikoganei branch (Wednesdays and Saturdays), and the Akatsuka branch (Tuesdays and Fridays). The three branches held two classes per day: one for Study 1, typical-volume (three-set) low-load resistance training, and the other for Study 2, low-volume (one-set) low-load resistance training. The three-set resistance training protocol has been recommended in several studies [31,32]. Each branch conducted four classes per week. The participants received exercise instructions in their assigned class for 12 weeks and were not allowed to attend any other classes. They took 24 resistance training classes twice weekly for 12 weeks.

The resistance training program consisted of four exercises: squats, split squats (right and left legs), push-ups, and crunches. All exercises involved the participants’ body weight. The order of exercises, number of repetitions per set, and rest time between sets are presented in Table 1.

**Table 1:** Training program.

A) Study 1: typical-volume training.												
Week	1–2		3–4		5–6		7–8		9–10		11–12	
Exercises	Order	Sets	Order	Sets	Order	Sets	Order	Sets	Order	Sets	Order	Sets
Squat	1	2	1	2	1	3	1	3	3	3	3	3
Split squat (right leg)	2	2	2	2	2	2	2	3	1	3	1	3
Split squat (left leg)	3	2	3	2	3	2	3	3	2	3	2	3
Push-up	4	2	4	2	4	3	4	3	4	3	4	3
Crunch	5	2	5	2	5	3	5	3	5	3	5	3
Repetitions per set	6		8		8		8		8		8	
CON-ECC (seconds)	3−3		3−3		3−3		3−3		3−3		3−3	
Interval (seconds)	60		60		60		60		60		60	
Frequency (days/week)	2		2		2		2		2		2	

B) Study 2: low-volume training												
Week	1−2		3−4		5−6		7−8		9−10		11−12	
Exercises	Order	Sets	Order	Sets	Order	Sets	Order	Sets	Order	Sets	Order	Sets
Squat	1	2	1	1	1	1	1	1	3	1	3	1
Split squat (right leg)	2	2	2	1	2	1	2	1	1	1	1	1
Split squat (left leg)	3	2	3	1	3	1	3	1	2	1	2	1
Push-up	4	2	4	1	4	1	4	1	4	1	4	1
Crunch	5	2	5	1	5	1	5	1	5	1	5	1
Repetitions per set	6		8		8		8		8		8	
CON-ECC (seconds)	3−3		3−3		3−3		3−3		3−3		3−3	
Interval (seconds)	60		60		60		60		60		60	
Frequency (days/week)	2		2		2		2		2		2	

CON-ECC; the time (seconds) in the concentric phase and eccentric phase of each repetition.

Interval; rest time between each set.

### 2.4 Test food

The LM extract used in these studies was commercially prepared (Lemon Myrtle UP™, Kaneka Corporation, Osaka, Japan). LM leaves were extracted using boiling water, and the extract was powdered with dextrin. The content of casuarinin, a type of ellagitannin, was determined according to the manufacturer’s specifications. The total weight of an LM tablet was 250 mg, which consisted of 1.25 mg of casuarinin derived from LM (Lemon Myrtle UP™) and excipients (crystalline cellulose, maltitol, silicon dioxide, and calcium stearate). The total weight of the placebo tablet was 250 mg, containing only excipients (crystalline cellulose, maltitol, silicon dioxide, and calcium stearate) and caramel color. The LM and placebo tablets were indistinguishable in appearance.

During the intervention period, each participant ingested two LM or placebo tablets daily with water for 12 weeks. The participants were instructed to take the assigned LM or placebo tablets in the morning and not miss any dose.

### 2.5 Body composition

Body composition, including body weight, fat mass, and percentage of body fat (% fat), was measured using bioelectrical impedance analysis with a body composition analyzer (InBody 270, InBody Japan Inc., Tokyo, Japan). BMI was calculated as body weight (kg) divided by height squared (m^2^).

### 2.6 Muscle thickness

The AT muscle thickness in each participant was measured using a B-mode ultrasound device with a 5–18 MHz scanning head (LOGIQ e, GE HealthCare Japan, Tokyo, Japan). The anterior aspect of the thigh (AT) was evaluated at the midpoint between the greater trochanter and the lateral condyle of the femur. The participants were required to rest in a sitting position for at least 30 min before the measurement and to remain in a supine position during the procedure. The measurements were conducted according to the method used in a previous study, the details of which were described in those studies. [33,34].

### 2.7 Physical functions

Physical function was evaluated through normal walking speed (10-m walk test, m/s), maximum walking speed (10-m walk test, m/s), and 30-second chair stand (CS-30, repetitions) test. Walking performance was assessed by timing each participant as they walked across a 10-m corridor on a hard-surfaced floor. The corridor width was set to 1 m. The participants performed two timed trials and were encouraged to maintain a straight course. They were instructed to walk down the corridor as quickly as possible. For the CS-30 test, the participants were instructed to complete the maximum number of sit-to-stand trials using a chair (40 cm height) with both arms crossed over the chest. Participants were instructed to sit on a 40-cm stool with both arms crossed against the chest and to stand up when their bottom touched the stool as many times as possible in 30 s, as outlined by Jones [35]. The test was initiated with the participant in a sitting position. The total number of stands was recorded as a score.

### 2.8 Brief-type self-administered diet history questionnaire

To accurately evaluate the effect of resistance training or active ingredients on changes in food intake during the study period, we administered the BDHQ, which is widely used in Japan [36,37]. The BDHQ is a four-page structured questionnaire that includes questions on the frequency of food intake and eating habits in a structured format during the preceding month, with a response time of approximately 15 min. Nutrient intake was calculated based on standard nutrient tables for food composition in Japan.

### 2.9 Safety assessment

The participants kept diaries recording their subjective symptoms, medications, test food intake, and lifestyle habits during the intervention period. The content of their diaries was reviewed, and participants were interviewed in detail every 4 weeks by the investigators to monitor their health status. Medical interviews, blood pressure measurements, pulse assessment, and blood tests were performed and recorded before and after the intervention.

### 2.10 Statistical analyses

Sample size calculations were based on a previous study by Sawada et al. (2024), which reported a similar intervention effect. For Studies 1 and 2, a priori calculations were performed, assuming an effect size of d = 0.8, 80% power, and a two-tailed significance level of 0.05. For Study 1, these calculations indicated that 26 participants per group were required, which was increased to 32–33 per group after accounting for an anticipated dropout rate of 20%. For Study 2, which involved a slightly smaller training set, the required sample size of 26 participants per group was adjusted for a 15% anticipated dropout rate, resulting in a final minimum of 30–31 participants per group.

All values are expressed as means ± standard deviations. To account for the repeated-measures structure of the data, mixed-effect regression models were fitted with fixed effects for group (LM or placebo), time (baseline, 6 weeks, and 12 weeks), and group-by-time interaction (equivalent to difference-in-differences estimates), and a random intercept for participants. This modeling approach served as the primary method of statistical inference, and effect sizes with 95% confidence intervals (CIs) were obtained for each outcome (primary and secondary). All main findings and conclusions were based on these mixed-effect regression results.

As supplementary analyses, statistical evaluation of each parameter before the intervention was performed using Fisher’s exact test or the unpaired t-test. Comparisons between the groups for percentage changes in AT muscle thickness (primary outcome), normal and maximum walking speeds, and CS-30 scores (secondary outcomes) were performed using a closed testing procedure to control for multiple comparisons. An independent t-test was conducted to compare the mean values between the two groups at 12 weeks. If a significant difference was found at 12 weeks (*p* < 0.05), an independent t-test was performed to compare the mean values at 6 weeks. The incidence of adverse events was evaluated using Fisher’s exact test.

Significance was set at *p* < 0.05. Statistical analyses were performed using Bell Curve for Excel (Social Survey Research Information Co., Ltd., Japan) and R statistical software (version 4.3.1; R Foundation for Statistical Computing, Vienna, Austria).

## 3 Results

**Fig. 1:**
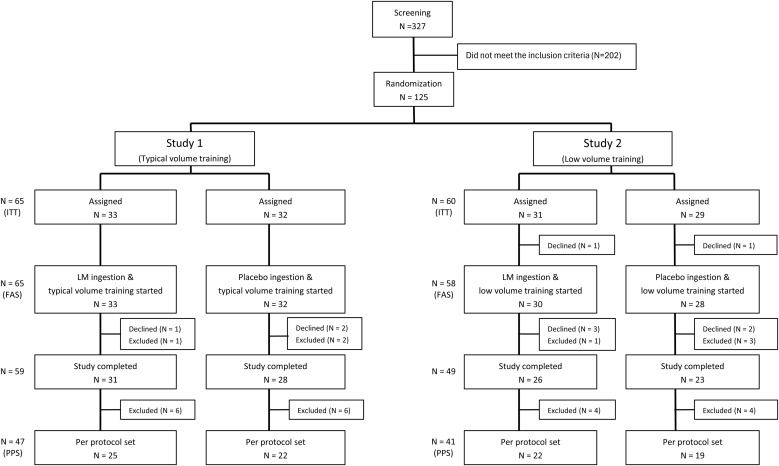
Flowchart showing the distribution of participants throughout the intervention.

The participant flowchart is shown in Fig. 1. In total, 327 older adults were screened, and 125 participants were judged eligible for these studies based on the investigators’ inclusion and exclusion criteria. They were randomized into two studies, each with two groups: LM + exercise for Study 1 (n = 33), placebo + exercise for Study 1 (n = 32), LM + exercise for Study 2 (n = 31), and placebo + exercise for Study 2 (n = 29). Details are shown in the flowchart in Fig. 1, and adverse events observed in Studies 1 and 2 are summarized in the supplementary table.

### 3.1 Before the intervention

The characteristics of the participants at baseline and before the intervention are shown in Table 2A and B, respectively.

**Table 2:** Baseline characteristics.

**A)** Baseline characteristics of the participants in Study 1			
	LM (n = 25)	Placebo (n = 22)	*P*-value^1^
***Participants (number) [Men:Women]***	25 [12:13]	22 [10:12]	1.000
***Physical parameters***			
Age (year)	70.5 ± 4.2	71.1 ± 5.1	0.652
Body weight (kg)	57.5 ± 11.0	56.9 ± 8.8	0.828
Height (cm)	160.9 ± 8.2	159.2 ± 9.0	0.507
BMI (kg/m^2^)	22.0 ± 2.8	22.4 ± 2.1	0.654
***Muscle thickness***			
Anterior thigh (mm)	27.55 ± 6.45	27.99 ± 4.73	0.794
***Physical functions***			
Normal walking speed (m/sec)	1.41 ± 0.18	1.43 ± 0.19	0.747
Maximum walking speed (m/sec)	1.83 ± 0.28	1.80 ± 0.22	0.750
CS-30 (times)	18.4 ± 4.15	19.3 ± 3.81	0.439
***Food intake***			
Energy (kcal/day)	1610 ± 343.9	1667 ± 503.5	0.650
Protein (g/day)	66.8 ± 18.0	73.2 ± 23.7	0.297
Fat (g/day)	57.5 ± 15.7	57.2 ± 18.3	0.951
Carbohydrates (g/day)	189.0 ± 58.0	200.0 ± 68.6	0.557

**B)** Baseline characteristics of the participants in Study 2			
	LM (n = 22)	Placebo (n = 19)	*P*-value^1^
***Participants (number) [Men:Women]***	22 [12:10]	19 [9:10]	0.885
***Physical parameters***			
Age (year)	71.7 ± 5.6	70.9 ± 4.2	0.616
Body weight (kg)	58.7 ± 11.2	59.4 ± 7.7	0.816
Height (cm)	160.6 ± 7.8	160.1 ± 9.4	0.853
BMI (kg/m^2^)	22.6 ± 3.0	23.1 ± 1.7	0.500
***Muscle thickness***			
Anterior thigh (mm)	27.06 ± 5.92	28.15 ± 6.22	0.568
***Physical functions***			
Normal walking speed (m/sec)	1.35 ± 0.20	1.44 ± 0.23	0.195
Maximum walking speed (m/sec)	1.80 ± 0.22	1.88 ± 0.25	0.291
CS-30 (times)	18.9 ± 2.99	18.5 ± 3.56	0.673
***Food intake***			
Energy (kcal/day)	1878 ± 683.7	1902 ± 728.3	0.915
Protein (g/day)	73.0 ± 31.4	75.1 ± 24.3	0.815
Fat (g/day)	60.9 ± 25.4	62.5 ± 25.8	0.844
Carbohydrates (g/day)	246.7 ± 107.7	244.7 ± 107.6	0.954

Values are presented as the means ± SD.

BMI, body mass index; CS-30, the 30-s chair stand test.

1 Comparison between groups using Fisher's exact test or unpaired Student's t-test.

No significant differences in age, body weight, height, or BMI were observed between the LM and placebo groups at baseline in either Study 1 or 2. Additionally, no differences in muscle thickness or physical function were observed between the groups at baseline in either Study 1 or 2. Furthermore, based on the BDHQ results, no significant differences in daily total energy, protein, fat, and carbohydrate intake were observed between the LM and placebo groups before the intervention.

### 3.2 After the intervention

**Fig. 2:**
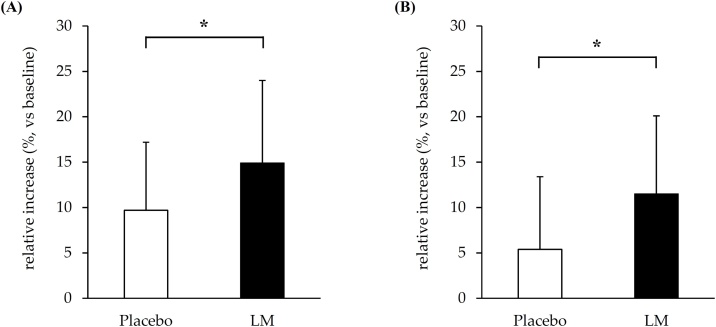
Effects of low-volume training and lemon myrtle (LM) administration on muscle thickness. The combination of LM ingestion and either (A) typical volume or (B) low-volume training significantly increased the relative increase in anterior thigh muscle thickness compared to the combination of placebo ingestion and training. Values are presented as the means ± SD. Comparison between groups using unpaired Student’s t-test (* p < 0.05).

For Study 1, mixed-effect regression analysis indicated greater increases in AT muscle thickness (primary outcome) in the LM group compared with the placebo group (*p* < 0.10 at all time points), with a 12-week difference of 1.29 mm (95% CI: −0.17 to 2.75 mm, *p* = 0.082). The relative increase from baseline was 14.9% (95% CI: 11.1–18.6%) vs. 9.7% (95% CI: 6.3–13.0%) (*p* < 0.05, Fig. 2A), with an effect size of 0.155. Muscle hypertrophy occurred in 92.0% of the participants in the LM group and 86.4% in the placebo group. No significant group-by-time interactions were observed for normal walking speed, maximum walking speed, or CS-30 score (secondary outcomes).

For Study 2, mixed-effect regression analysis indicated a significant difference in AT muscle thickness (primary outcome) between the LM and placebo groups, with a 12-week difference of 1.59 mm (95% CI: 0.19–2.98 mm, *p* < 0.05). The relative increase from baseline was 11.5% (95% CI: 7.7–15.3%) vs. 5.4% (95% CI: 1.5–9.3%) (*p* < 0.05, Fig. 2B), with an effect size of 0.745. Muscle hypertrophy occurred in 90.9% of the participants in the LM group and 73.7% in the placebo group. No significant group-by-time interactions were found for normal walking speed, maximum walking speed, or CS-30 score (secondary outcomes).

Dietary intake (energy, protein, fat, carbohydrate) did not differ significantly between groups before or after the intervention (BDHQ survey). Detailed values are presented in Table 3A and B.

**Table 3:** Effects of training and LM intake on muscle thickness and physical functions.

**A)** Effects of typical-volume training and LM intake on muscle thickness and physical functions in Study 1				
	Time	LM (n = 25)	Placebo (n = 22)	*P*-value^1^
***Muscle thickness***				
Anterior thigh (%, vs baseline)	6 weeks	4.9 ± 7.1	0.9 ± 9.4	0.102
	12 weeks	14.9 ± 9.1	9.7 ± 7.5	0.039
***Physical functions***				
Normal walking speed (%, vs baseline)	6 weeks	5.0 ± 9.9	6.3 ± 11.4	–
	12 weeks	6.2 ± 11.1	4.6 ± 11.2	0.635
Maximum walking speed (%, vs baseline)	6 weeks	5.2 ± 7.7	7.0 ± 9.5	–
	12 weeks	7.2 ± 8.0	6.8 ± 10.2	0.902
CS-30 (%, vs baseline)	6 weeks	10.5 ± 16.7	15.5 ± 16.4	–
	12 weeks	19.1 ± 19.4	26.7 ± 19.8	0.191

**B)** Effects of low-volume training and LM intake on muscle thickness and physical functions in Study 2				
	Time	LM (n = 22)	Placebo (n = 19)	*P*-value^1^
***Muscle thickness***				
Anterior thigh (%, vs baseline)	6 weeks	5.7 ± 8.4	1.3 ± 9.7	0.123
	12 weeks	11.5 ± 8.6	5.4 ± 8.0	0.026
***Physical functions***				
Normal walking speed (%, vs baseline)	6 weeks	9.2 ± 9.6	2.5 ± 10.5	–
	12 weeks	8.7 ± 14.2	2.3 ± 10.0	0.107
Maximum walking speed (%, vs baseline)	6 weeks	4.5 ± 9.5	4.7 ± 15.2	–
	12 weeks	5.2 ± 7.9	8.3 ± 22.9	0.564
CS-30 (%, vs baseline)	6 weeks	11.2 ± 16.1	18.8 ± 15.5	–
	12 weeks	22.0 ± 18.7	26.4 ± 17.4	0.451

Values are presented as the means ± SD.

CS-30, the 30-s chair stand test.

### 3.3 Safety assessment

In both studies, the number of adverse events during the 12-week intervention period was similar between the two groups (Study 1: 14 in the LM group, nine in the placebo group; Study 2: 14 in the LM group, nine in the placebo group), and no significant differences were observed. Additionally, the physicians judged all adverse events to be unrelated to the test food.

## 4 Discussion

The two independent randomized controlled trials described in this article investigated the effects of LM supplementation, in addition to different volumes of low-load bodyweight resistance exercise, on muscle thickness (primary outcome) and physical function (secondary outcomes). In Study 1, when compared to placebo + exercise, LM combined with three sets of low-intensity resistance exercise had no significant effects on the outcome measures, although a statistical trend was found favoring LM + exercise group on increased anterior thigh muscle thickness. In Study 2, when compared to placebo + exercise, LM combined with one set of low-intensity resistance exercise led to improvements on muscle thickness, but not on physical function.

On the other hand, in Study 2, a lower-volume (one-set) low-load resistance training intervention was conducted with participants who were aware that the decline in muscle strength had worsened (total n = 41, 22 in LM and 19 in placebo groups). As a result, analyses focusing on percentage change comparisons between the two groups, effect size evaluations, and mixed-effect regression models, all demonstrated that the LM group had a significantly greater training effect on the increase in AT muscle thickness compared with the placebo group. Regarding the other parameters of physical function, no significant differences were observed between the LM and placebo groups. The percentage change rate of AT in the LM group was approximately 12%, which is nearly the same as the value of previous studies with larger training volume [29,33,34]. Therefore, LM intake appears to have compensated for the reduced training volume.

In these studies, three sets of training took approximately 30 min, while one set of training took only approximately 10 min. The combination of low-volume training and LM intake could improve time efficiency in training, which is valuable for continuing training in daily life. This could become an effective and efficient method against sarcopenia and frailty, potentially reducing the risks and burden associated with increased exercise volume and time. The conventional nutritional strategy for countermeasures against sarcopenia and frailty involves ensuring sufficient protein intake and resistance exercise. In Study 2, LM supplementation appeared to enhance muscle hypertrophy in the context of low-volume resistance training; however, no improvements were observed in physical function parameters (walking speed and CS-30). These findings suggest that while LM intake may contribute to the preservation of muscle mass as a preventive strategy against sarcopenia, regular and sufficient training remains essential for maintaining and improving physical function.

Setting suitable training variables is important for muscle hypertrophy. Regarding training frequency, although many studies have employed a higher frequency (3–7 days per week) [[38], [39], [40], [41], [42]], our recent studies [33,34] showed that low-load resistance training using participants’ body weights only twice a week induced muscle hypertrophy in older participants. As mentioned in the Methods section, our pilot study [29] demonstrated that typical-volume (three-set) low-load resistance training using body weight significantly increased muscle size despite reducing the number of exercises and repetitions. The combination of this training and LM leaf extract resulted in a significantly greater increase in muscle size than resistance training with a placebo condition in 14 older men and women (seven per group) [29]. However, when physical and mental frailty progress and appetite decreases, especially in older adults, it may be difficult to increase their protein intake. Additionally, restricting protein intake in patients with chronic kidney disease may be effective in slowing the progression of the disease [[43], [44], [45], [46]]. Therefore, there are cases in which sufficient protein intake cannot be ensured for clinical reasons. The intake of LM, which was the focus of the present study, aims to enhance muscle hypertrophy and represents a significantly different approach from conventional nutritional strategies. Furthermore, these benefits were achieved with a daily intake of two 250 mg tablets, each containing 1.25 mg of casuarinin derived from LM. This is a provisional intake, and even smaller doses may be effective. However, further studies are required. Additionally, no adverse events related to LM intake were reported in the present study, at least at the dose used, which appears to be safe for humans.

Taken together, LM intake may enhance muscle hypertrophy when combined with low-load resistance training, particularly under low-volume conditions. LM may have limited utility in populations who can perform higher volumes of training, but it may be of interest in populations with restricted exercise capacity. However, the clinical relevance of these findings remains to be established, and further large-scale studies are warranted.

These studies have some limitations. First, it did not examine the effects of LM consumption alone. These studies showed that LM intake enhances the training effect; however, the effect of LM intake without training on skeletal muscles is unknown. This hypothesis warrants further investigation. Second, because these studies were conducted on older individuals who were aware of muscle strength decline, it remains unclear whether LM intake enhances muscle hypertrophy in other people (e.g., healthy older adults, young people, or patients with chronic kidney disease). The relative load in body weight-based resistance training on working muscles is associated with the ratio of body mass to lower body strength. Therefore, muscle hypertrophy induced by this type of training may be more favorable in older women who generally have a lower relative ratio of lower extremity muscle strength to body mass [47]. Consequently, the effects of bodyweight training may vary significantly depending on the participant, and these differences in characteristics may need to be considered when assessing the effects of LM ingestion. Further studies are needed to address these limitations.

## 5 Conclusions

LM intake may enhance anterior thigh muscle hypertrophy in older adults with self-reported muscle strength decline after 12 weeks of low-load resistance training. The effect was particularly evident under low-volume (one-set) training, whereas no statistically significant improvement was confirmed under typical-volume (three-set) training. These findings suggest that LM may be beneficial for populations with limited capacity for exercise, although its clinical relevance remains to be established.

## CRediT authorship contribution statement

Study concept, A.N., S.H., Y.T., and S.M.; study design, S.S., H.O., and S.M.; test supplements, A.N., S.H., and Y.T.; study experiments, S.S., Shi.M., H.O., and S.M.; data analysis, S.S., H.O., and S.M.; result interpretation, S.S., H.O., and S.M.; ﬁgure preparation, S.S. and S.M.; manuscript drafting, S.S., H.O., and S.M.; editing and revision of the manuscript, S.S., A.N., S.H., Shi.M., H.O., and S.M.; approval of the ﬁnal version of the manuscript, S.S., A.N., S.H., Y.T., Shi.M., H.O., and S.M.

## Informed consent statement

Appropriate explanations were provided to the participants regarding important factors, including the study purpose, content, methods, and predicted adverse reactions. Informed consent was obtained from all the participants during the writing process. The current our studies were registered in the UMIN Clinical Trials Registry (UMIN 000050432).

## Institutional review board statement

All the experiments conformed to the principles of the Declaration of Helsinki. The study protocol and related documents were approved by the Ethics Committee of the Juntendo University Graduate School of Health and Sports Science Institutional Review Board (Approval Number: 2022-131).

## Declaration of Generative AI and AI-assisted technologies in the writing process

We have not used any AI at all.

## Funding

This research was funded by Kaneka Corporation (Osaka, Japan).

## Data availability statement

Data supporting the findings of these studies are available upon request from the corresponding author.

## Declaration of competing interest

AN, SH, and YT are employees of Kaneka Corporation (Osaka, Japan), which funded these studies. They were responsible for the test supplements, but were not involved in the investigation, formal analysis, or data curation. The results of these studies are clear and without fabrication, falsification, or inappropriate data manipulation.
